# The biomechanics of piano playing: a systematic review of kinematic, kinetic, and electromyographic literature

**DOI:** 10.3389/fpsyg.2025.1690422

**Published:** 2026-01-05

**Authors:** Aljoša Jurinić, Marija Pranjić, Aiyun Huang, Timothy A. Burkhart, Daphne Tan, Praneeth Namburi

**Affiliations:** 1 Faculty of Music, University of Toronto, Toronto, ON, Canada; 2 Institute for Medical Engineering and Science, Massachusetts Institute of Technology, Cambridge, MA, United States; 3 Developmental Medicine, Boston Children’s Hospital, Harvard Medical School, Boston, MA, United States; 4 Faculty of Kinesiology and Physical Education, University of Toronto, Toronto, ON, Canada; 5 MIT.nano Immersion Lab, Massachusetts Institute of Technology, Cambridge, MA, United States

**Keywords:** piano, biomechanics, piano pedagogy, piano technique, kinematics, kinetics, electromyography

## Abstract

Piano playing is one of the most complex human activities, involving an intricate interplay between the cognitive, neural, and musculoskeletal systems. Understanding the biomechanics of piano playing could have important implications for evidence-based pedagogy, optimizing skill acquisition, increasing practice efficiency, and minimizing the risk of performance-related injuries. This systematic review synthesizes existing literature in piano biomechanics. A comprehensive search across five databases (MEDLINE, PsycINFO, SCOPUS, Music Index, and ERIC) yielded 7,671 studies, of which 53 met inclusion criteria. These studies utilized kinematic, kinetic, and electromyographic measurements during piano performance under varying task conditions, including isolated keystrokes, novel excerpts, self-selected repertoire, and standard piano literature. The results were synthesized to address (1) how variations in pianistic technique (e.g., type of touch, finger independence) influence kinematics, kinetics, and muscle activation, and (2) how task demands (e.g., tempo, loudness), and performance demands (e.g., fatigue, ergonomics) affect pianists’ biomechanical characteristics. Together, current biomechanical evidence indicates that touch type, finger independence, intersegmental kinematics, and muscular activation are modulated by pianists’ skill level, anthropometry, task demands, and muscular fatigue. We further discuss and link the existing literature to pedagogy, practice, and performance, thereby demonstrating that biomechanical parameters are not merely abstract descriptors of motion but are integral to pedagogy, musical expression, and sustainable performance. While there is an accumulating wealth of biomechanical data involving piano playing, its integration into standardized pedagogy remains limited. Interdisciplinary collaboration in piano biomechanics is therefore essential to advance our understanding of musical expression, communication, and wellbeing, ultimately supporting the development of sustainable playing techniques that can be effectively translated into pedagogical frameworks.

**Systematic review registration:** Unique Identifier: 10.17605/OSF.IO/TSNY8

## 1 Introduction

Piano playing is an expression of human creativity, culture, spirit, and emotions, whose artistic values have been widely discussed in scholarly and popular literature (e.g., Loesser, 2012). In addition to these well-documented psychosocial, affective, and cognitive aspects of music making, piano playing is considered one of the most complex sensorimotor activities (Parsons et al., 2005) and has been described as “the ultimate example of elite performance in complex hand dexterity” (Metcalf et al., 2014). Thus, the utilization of the performer’s body is an indispensable aspect of piano playing. Despite this, piano pedagogy remains primarily based on tradition, intuition (James, 2012), and the idiosyncratic practices of concert pianists and teachers that are rarely rooted in scientific understanding of the underlying biomechanics (Takamizawa and Kenway, 2023).

Pianists have also been described as “athletes of the hand” (Davidson, 2017), as they engage in extensive sensorimotor training from an early age which comprises skill acquisition, rhythmic coordination, perceptual acuity, and artistic control and expression. Similar to athletes, the prevalence of performance-related injuries is a significant issue among pianists, with studies indicating that more than 60% of active pianists experience muscular discomfort, and some develop serious medical conditions such as tendonitis and focal dystonia (Bruno et al., 2008). In contrast to predominantly acute injuries in athletes, playing-related musculoskeletal disorders (PRMDs) of pianists are mostly chronic, making them less noticeable until performance is significantly affected (McCrary et al., 2023).

Nowadays, pianists face additional challenges considering that the concert halls are larger than in previous centuries requiring greater sound intensity, the keys on the contemporary pianos are heavier than on their predecessors, and the competitiveness and repertoire demands have significantly grown over the last several decades (Bokiau et al., 2017). For instance, the number of international piano competitions surged from 30 in 1965 to 330 in 2010 (Alink, 2010), with the rise of online competitions further accelerated by the COVID-19 pandemic (Tokay, 2023). Together, these increasing demands on piano performance necessitate a rigorous, individualized, and scientifically informed pedagogical approach to skill acquisition and injury risk reduction.

Owing to the exceptional motor and cognitive demands of piano playing, pianists have served as model subjects in neuroscience, providing key evidence on how complex motor skills are represented, refined, and adapted in the brain. Characterized by precision, bilateral coordination, and expressive control, piano playing offers an ideal framework for linking biomechanical processes with neural processes underlying expert motor behavior (e.g., Lotze et al., 2003; Parsons et al., 2005).

Biomechanics, the application of the mechanical laws to the movement or structure of living organisms, is a valuable tool for analyzing the body’s role in piano playing. By investigating the musculoskeletal mechanisms that underlie musical performance, new insights on the performer’s movement quality can be gained to facilitate healthier and more sustainable playing strategies. Specifically, kinematic measurements quantify the spatial and temporal aspects of motion without taking into consideration the forces causing it, while kinetic measures quantify the forces and torques that drive the motion (Özkaya et al., 2012). In the context of piano playing, these parameters allow researchers to quantify the motion of body segments and compute the forces that are exerted. In addition, electromyography (EMG) is used to capture electrical signals from the muscle, which can identify the muscles involved in specific movements. Parameters derived from EMG signals, such as the median frequency, can serve as indicators of muscular fatigue (Achmad et al., 2022).

Over the past decade, studies have increasingly investigated movement patterns in musical performance, including systematic reviews of the biomechanical methods in woodwind (López-Pineda et al., 2023) and brass instrumentalists (Herrmann et al., 2021), posture in musicians (Rousseau et al., 2023), and ergonomics for pianists and violinists (Chi et al., 2020). Additionally, non-systematic literature reviews covering various aspects of the biomechanics of piano playing have been conducted (Furuya and Altenmüller, 2013; Goebl, 2017; James, 2018). Despite these recent advances in scientific literature, there is a profound gap between research findings and their practical implications into music pedagogy (MacRitchie, 2015). More recently, a systematic review by Mailly et al. (2025) examined the influence of expressive parameters on pianists’ kinematics and muscle activity.

To date, however, no work has systematically synthesized the broader body of piano biomechanics research by mapping and critically appraising the existing evidence on pianistic technique. The current review aims to fill this knowledge gap by addressing the following questions: (1) How do variations in pianistic technique (e.g., type of touch, finger independence) influence kinematics, kinetics, and muscle activation? (2) How task demands (e.g., tempo, loudness), and performance demands (e.g., fatigue, ergonomics) affect pianists’ biomechanical characteristics.

## 2 Methods

The literature search was performed in compliance with the Joanna Briggs Institute methodological framework (Munn et al., 2020), following the Preferred Reporting Items for Systematic Reviews and Meta-Analyses (PRISMA) guidelines (Hutton et al., 2015). The protocol was registered with the Open Science Framework on April 20, 2024, and is available online at https://osf.io/nrqma/.

### 2.1 Information sources and search strategy

The search was carried out across five major databases, including MEDLINE, PsycINFO, SCOPUS, Music Index, and ERIC for articles published before April 1, 2024. The PEO (Population, Exposure, Outcomes) mnemonic was used to extract the data and formulate the search strategy (i.e., Population = Pianists; Exposure = Piano playing; Outcomes = Biomechanical measurements [kinematics, kinetics, and electromyography]). Subject headings were adapted for each database. Additionally, we examined reference lists of included studies and screened any relevant publications. Table 1 provides the search strategy performed in MEDLINE (see Supplementary Table S1 for the full electronic search strategy).

**Table 1:** Search strategy performed in MEDLINE.

Database	MEDLINE (Ovid)		
Limits	Language: “English,” Publication Type: “Article”		
Search Query	Population, Exposure	AND	Outcomes
	Pianists / Piano Playing		Biomechanics
	Keywords piano.mp. OR (music* adj3 perform*).mp. OR pianist*.mp.		Keywords (biomechanic* or movement* or muscle* or electromyograph* or motor* or limb* or motion* or mechanic* or kinematic* or kinetic*).mp
Results	1242		

### 2.2 Eligibility criteria

Articles were included if they: (1) involved pianists with an advanced level of training, (2) involved piano playing tasks on a regular-sized piano keyboard, (3) included biomechanical measurements during playing, (4) were published in English, and (5) were categorized as original research. Studies were ineligible if they: (1) included only inexperienced players, (2) involved tasks that did not capture piano playing (e.g., finger-tapping paradigms), (3) measured only posture, and (4) were review papers, book chapters, single-case studies or conference proceedings.

### 2.3 Screening and data extraction

All titles, abstracts, and full-text publications were screened by two independent reviewers (AJ and MP) for inclusion criteria; any discrepancies were resolved through discussion. The appropriate screening and filtering processes of the search results were done via Covidence software (Veritas Health Innovation, Melbourne, Australia). Relevant studies were further evaluated and inconsistencies among the reviewers regarding study selection were discussed and resolved by consensus. The two reviewers systematically and independently extracted the following information from the included papers using a data extraction form: publication details, sample characteristics (i.e., age range, sex, sample size, inclusion criteria), study design, tasks, outcome measures, and key findings. One reviewer (AJ) screened all of the eligible studies and verified the correctness of the extracted information. Meta-analyses could not be undertaken due to the heterogeneity of screening tools, tasks, functional domains, and outcome measures. As part of data extraction, each study was assigned to a thematic category according to its primary research focus (see Table 2). This categorization served as the framework for the subsequent synthesis of findings, in line with PRISMA guidance on structured reporting.

**Table 2:** Key characteristics and critical appraisal of included studies.

Author(s)	Sample (*N*)	Age (M ± SD)	Task	Piano	Measurement	Anatomical region(s)	Key findings	Quality	Referred in section(s)
Aranceta-Garza et al. (2021)	16	24 ± 11	Scale at three tempi, existing literature	Grand	EMG	Torso	Chair with lumbar support ↓ erector spinae muscles activity and ↑ personal comfort	High (7)	3.7.2.
Baeyens et al. (2020)	10	23.9	Self-selected (fast and slow)	NR	EMG	Forearm	Fast excerpt ↑ extensor carpi radialis activity, no change in median EMG frequency over time	Average (5)	3.7.1.
Baeyens et al. (2022)	10	23.9	Self-selected (fast and slow)	NR	EMG	Shoulder	Fast excerpt ↑ trapezius activity and ↓ median EMG frequency over time	Average (5)	3.7.1.
Bernardi et al. (2013)	24 (MP = 8, PP = 8, C = 8)	MP = 30 ± 10 PP = 31 ± 9 C = 32 ± 9	Existing literature (metronome, RH)	Digital	Kinematics, MIDI	Wrist, hand (R)	Movement velocity and timing improvements: Physical > Mental > No Practice	High (8)	
Chung et al. (1992)	9 (WPT = 5, TPT = 4)	22–50	Novel excerpts, existing literature (metronome)	Upright	Kinematics	Wrist	Wrist ROM ↑ trills and arpeggios; “weight playing” technique ↓ wrist ROM than “traditional” playing	Poor (4)	
Dalla Bella and Palmer (2011)	4	24	Novel excerpts (metronome before playing, RH)	Digital	Kinematics	Hand (R)	↑ tempo = ↑ peak finger heights and ↑ loudness	High (7)	3.6.1.
Degrave et al. (2020)	12	33.3 ± 4.7	Isolated keystrokes (metronome, RH)	Grand	EMG, kinematics	Shoulder, torso, upper limb (R)	Pressed touch ↑ triceps brachii; Struck touch ↓ anti-gravity muscles; *Staccato* ↑ muscular activity of shoulder muscles	High (7)	3.4.1.
Engel et al. (1997)	5 (*E* = 2, A = 2, *N* = 1)	NR	Existing literature	Digital	Kinematics, MIDI	Wrist, hand (R)	“Thumb-under” maneuver = anticipatory modifications	Average (5)	3.4.2.
Ferrario et al. (2007)	19 (*E* = 8, S/T = 11)	CP = 44.8 ± 15 S/T = 24.4 ± 14.8	Existing literature	Grand	Kinematics, kinetics (derived)	Wrist, hand (R)	Concert pianists ↑ movements of non-striking fingers	Average (6)	3.4.2.
Furuya and Kinoshita (2007)	14 (*E* = 7, *N* = 7)	*E* = 24.3 ± 3.2 *N* = 21 ± 4.6	Isolated octaves (four LL, RH)	Upright	Kinematics	Shoulder, pelvis, upper limb (R)	Experts = proximal-to-distal temporal relationship of the peak angular velocity, maintained as loudness ↑	High (7)	3.5., 3.6.2.
Furuya and Kinoshita (2008a)	14 (*E* = 7, *N* = 7)	*E* = 24.3 ± 3.2 *N* = 21 ± 4.6	Isolated octaves (four LL, RH)	Upright	Kinematics, kinetics	Shoulder, elbow, wrist, hand (R)	Experts ↑ motion-dependent interactions; Novices ↑ muscular torque; ↑ loudness ↑ muscular torque: experts ↑ proximal, novices ↑ distal joints	High (7)	3.5., 3.6.2.
Furuya and Kinoshita (2008b)	16 (*E* = 8, *N* = 8)	*E* = 24 ± 3 *N* = 20.6 ± 4.4	Isolated octaves (four LL, RH)	Upright	EMG, kinematics, kinetics (derived)	Shoulder, upper limb (R)	Experts ↑ flexed shoulder, wrist, MCP joints, ↓ key-force torque at the MCP joint, when ↑ loudness also ↑ muscle coactivation, but ↓ than novices	High (7)	3.5. + 3.6.2.
Furuya et al. (2009)	14 (*E* = 7, *N* = 7)	*E* = 24.3 ± 3.2, *N* = 21 ± 4.6	Isolated octaves (four LL, RH)	Upright	EMG, kinetics (derived)	Shoulder, upper limb (R)	Experts ↓ triceps & biceps activation → minimal ↑ with loudness; Novices ↑ triceps activation → further ↑ with loudness	High (7)	3.5. + 3.6.2
Furuya et al. (2010)	7	24.3 ± 3.2	Isolated keystrokes, (two LL, RH)	Upright	Kinematics, kinetics (derived)	Shoulder, elbow, wrist, hand (R)	Pressed touch ↑ shoulder and finger flexion velocity; Struck touch ↑ elbow extension velocity	Average (6)	3.4.1.
Furuya et al. (2011a)	10 (*E* = 5, A = 5)	*E* = 24.3 ± 3.2, A = 22.6 ± 1.1	Tremolo sixths (metronome, six tempi, RH)	Digital	EMG, kinematics, MIDI	Upper limb (R)	↑ Tempo = in experts ↓ thumb and fifth finger flexion velocity, ↓ extrinsic finger muscles activity, ↑ elbow velocity	High (8)	3.5. + 3.6.1.
Furuya et al. (2011b)	5	33 ± 8	Existing literature (30 excerpts from 11 pieces, metronome)	Digital	Kinematics, MIDI	Hand (R)	Non-striking fingers: ↑ consistency in thumb keystrokes, ↓ consistency in other fingers’ keystrokes	Average (6)	3.4.2.
Furuya et al. (2012)	18	30.2 ± 7.8	Isolated sixths (metronome, four tempi and LL, RH)	Upright	EMG, kinematics	Shoulder, upper limb (R)	↑ Loudness and tempo = ↓ elbow velocity, but ↑ velocity of the shoulder, wrist, and finger joints and ↑ muscular activity	Average (6)	3.6.1. + 3.6.2.
Furuya and Soechting (2012)	5	33 ± 8	Existing literature (30 excerpts from 11 pieces, metronome)	Digital	Kinematics, MIDI	Hand (R)	No effect of tempo on independent finger movements and rhythmic accuracy	High (7)	3.4.2.
Furuya and Yokota (2018)	27 (Rest = 9, NP = 9, VP = 9)	Rest = 23.4 ± 4.8 NP = 26 ± 8.4 VP = 23.8 ± 5.9	Existing literature (metronome, RH)	Digital	EMG, MIDI	Hand (R)	VP ↓ muscular activity and simultaneous activations across muscles; both NP and VP ↑ maximal tempo	High (7)	3.7.1.
Goebl and Palmer (2008)	12	27	Novel excerpts (four tempi, RH)	Digital	Kinematics, MIDI	Hand (R)	↑ Finger-key landmarks at faster tempi	High (7)	3.6.1.
Goebl and Palmer (2013)	12	27.7	Novel excerpt (10 tempi, metronome, RH)	Digital	Kinematics, MIDI	Wrist, hand (R)	Vertical fingertip motion (MCP joint flexion) = ↑ efficiency and ↑ temporal accuracy across tempi	High (7)	3.6.1.
Goubault et al. (2021)	49	SD = 27.4 ± 8.8 LD = 29.8 ± 8.6	Existing literature (metronome, RH)	Grand	EMG	Forearm (R)	Repetitive tasks ↑ fatigue in forearm muscles, especially extensors, ↑ velocity variability and ↓ note accuracy	High (7)	3.7.1.
Goubault et al. (2023)	49	SD = 27.4 ± 8.8 LD = 29.8 ± 8.6	Existing literature (metronome, RH)	Grand	EMG, kinematics	Shoulder, torso, pelvis, upper limb (R)	EMG variability ↑ in SD; LD group ↑ thorax variability (dexterous task); ↑ wrist variability (chord task)	High (7)	3.7.1.
Grieco et al. (1989)	6	NR	Existing literature (numerous excerpts)	Upright	EMG	Shoulder, torso, upper limb (R)	↓ Biceps and deltoid activity, ↑ forearm muscles activity; Females ↑ activity of trunk and shoulder muscles	Average (5)	
Honarmand et al. (2018)	10	25.4 ± 5.2	Self-selected piece	NR	EMG	Torso	Chair with a backrest ↓ muscular activity of the lumbar erector spinae muscles and ↑ personal comfort	Poor (4)	3.7.2.
Kaufman-Cohen et al. (2018)	15	21.72 ± 4	Existing literature	NR	Kinematics	Elbow, wrist (R)	MSD in the past 12 months = extreme wrist extension, elbow flexion, and smaller hand span; ↑ amount of practic*e* = ↑ MSD	Average (6)	
Kinoshita et al. (2007)	10	21.6 ± 1.7	Isolated keystrokes (very fast, varied LL, RH)	Upright	Kinetics	Hand (R)	Struck touch ↑ initial force and more fluctuations, but ↓ maximal force and impulse at *fortissimo* playing	High (7)	3.4.1., 3.6.2
Lai et al. (2015)	20 (LHS = 10, SHS = 10)	23.6 ± 6.3	Existing literature	Digital	Kinematics, kinetics	Hand	SHS ↑ inter-finger abduction and ↑ wrist ROM than LHS	High (8)	
Lai et al. (2023)	20 (*E* = 10, *N* = 10)	*E* = 20.3 ± 1.6 *N* = 22.4 ± 2.3	Isolated octaves and chords (metronome)	Upright	Kinetics	Hand	Experts ↑ consistent force of the thumb and fifth finger	High (8)	3.5.
MacRitchie et al. (2013)	9	NR	Existing repertoire	Digital	Kinematics, MIDI	Head, shoulder, torso, upper limb	Pianists’ global motion profiles reflected phrasing and harmonic structure	High (7)	3.6.3.
Massie-Laberge et al. (2019)	10	29.6 ± 5.8	Existing repertoire	Digital	Kinematics, MIDI	Head, shoulder, torso, upper limb, pelvis	Head quantity of motion was the most sensitive parameter differentiating expressive conditions	High (7)	3.6.3.
McCrary et al. (2023)	14	25.6 ± 2.7	Self-selected (three pieces)	NR	EMG	Shoulder, upper arm	10 min of playing = no evidence of fatigability in the trapezius, biceps, and triceps muscles	Average (5)	3.7.1.
Moore (1992)	4	NR	Trills	Grand	EMG, kinematics, MIDI	Forearm (R)	In constant tempo: ↑ in loudness = ↑ in MCP joint ROM, acceleration, EMG activity, and note duration	Poor (4)	3.6.2.
Nakahara et al. (2010)	9	24.0 ± 4.5	Existing repertoire	Upright	Kinematics	Torso, Upper Limb	Expressive playing = greater trunk and arm excursions aligned with musical phrasing	High (7)	3.6.3.
Oikawa et al. (2011)	28 (*E* = 14, *N* = 14)	*E* = 19.6 ± 1.4 *N* = 21.4 ± 1.5	Isolated octaves (metronome, three LL, RH)	Upright	EMG, kinematics	Forearm, Wrist (R)	↑ Loudness = ↑ muscular activity; wrist in a neutral position = ↓ extensor and flexor activity compared to flexed or extended	High (7)	3.6.2.
Oku and Furuya (2017)	24 (*E* = 16, *N* = 8)	*E* = 30.2 ± 7.8 *N* = 24.2 ± 9.1	Isolated sixths (metronome, four tempi and LL, RH)	Upright	Kinetics	Hand (R)	Experts ↓ peak force and impulse across tempi and ↑ consistency of force in slow tempi than novices	High (8)	3.5., 3.6.1. 3.6.2.
Parlitz et al. (1998)	20 (*E* = 10, A = 10)	*E* = 23, A = 29	Existing literature (metronome, RH)	Grand	Kinetics	Hand (R)	Amateurs ↑ force and impulse than experts at the same tempo and loudness; ↑ excerpt difficulty = ↑ force	High (7)	3.5.
Sakai et al. (1996)	10 (*E* = 5, A = 5)	29.1	Isolated repeated chords and a scale (metronome)	Digital	Kinematics	Wrist, Hand (R)	Scales ↑ MCP joint flexion; Chords ↑ wrist flexion	Average (6)	
Sforza et al. (2003)	5	9–70	Scale (metronome, three tempi)	Digital	Kinematics	Hand (R)	Finger motion repeatability ↑ in teachers/students than concert pianists	High (7)	
Sugawara (1999)	3	22.7	No details (duration = 5 min)	NR	Kinematics	Wrist	Pianists ↑ ulnar deviation than other instrumentalists	Poor (3)	
Thio-Pera et al. (2022)	8	23–41	Sequential octaves, existing literature, improvisation	Upright	EMG, kinematics	Torso, upper limb	Dexterous task ↑ activity of ventral forearm muscles (flexors); Octaves ↑ in the dorsal region (extensors)	High (7)	
Thompson and Luck (2012)	8	24.6	Existing literature	Digital	Kinematics	Head, shoulder, torso, upper limb	↑ Expression = ↑ head and torso movements	High (7)	3.6.3.
Tominaga et al. (2016)	7	21–39	Novel excerpt (RH)	Digital	Kinematics, MIDI	Hand (R)	↑ Rhythmic inconsistency = ↑ variability in striking and non-striking fingers; ↑ Loudness inconsistency = ↑ variability in striking fingers only	High (7)	3.4.2.
Turner et al. (2021)	2	NR	Scale (five tempi, metronome)	Grand	Kinematics	Shoulder, torso, upper limb	Faster tempo (from 8 notes/s) = changes in motor behavior strategies (note: 2 subjects only)	Average (6)	3.6.1.
Turner et al. (2022)	3	NR	Existing literature (three tempi, metronome)	Grand	Kinematics	Torso, hand (R)	Anthropometry and expertise influence trunk ROM	Average (6)	3.5.
Turner et al. (2023)	9	32.8 ± 3.7	Sixths in leaps (two tempi, metronome, RH)	Grand	Kinematics	Shoulder, torso, pelvis, upper limb (R)	Slow tempo ↑ variability of the wrist and elbow joints; ↑ Trunk motio*n* = ↑ elbow and shoulder variability	High (7)	3.6.1.
Verdugo et al. (2020)	9	34 ± 4.4	Isolated keystrokes (metronome, RH)	Grand	Kinematics	Torso, pelvis, upper limb (R), lower limb (L)	Struck touch ↑ elbow velocity; *Staccato* ↑ shoulder and wrist joint contributions; ↑ Trunk motion = ↑ segmental velocities	High (7)	3.4.1.
Verdugo et al. (2022)	9	32.8 ± 3.7	Isolated keystrokes (metronome, RH)	Grand	Kinematics	Torso, pelvis, upper limb (R), lower limb (L)	Pressed-*staccato* keystroke = proximal-to-distal sequencing	High (7)	3.4.1.
Winges et al. (2013)	10 (*E* = 4, A = 6)	37 ± 12	Existing literature (14 excerpts from 11 pieces, metronome)	Digital	EMG, MIDI	Forearm, hand (R)	The nature of the preceding and subsequent keystrokes affects the balance of EMG amplitudes across muscles	High (7)	3.4.2.
Winges and Furuya (2015)	10 (*E* = 5, A = 5)	33 ± 10	Existing literature (14 excerpts from 11 pieces, metronome)	Digital	Kinematics, MIDI	Hand (R)	Experts = broader range of strategies, i.e., ↑ movement covariation in some sequences and ↓ in others than amateurs	High (7)	3.4.2., 3.5.
Wolf et al. (1993)	8	NR	Existing literature	Digital	Kinetics (derived), MIDI	Hand (R)	More curved fingers ↓ tendon and joint forces compared to more extended fingers	Average (5)	
Yoshie et al. (2008)	12	21.9 ± 3.3	Novel excerpt (metronome)	Digital	EMG, MIDI	Shoulder, upper limb (R)	Evaluation condition ↑ EMG activity of all investigated muscles; ↑ EMG activity = ↑ key velocities	Average (5)	3.7.3.
Yoshie et al. (2009)	18	26.7 ± 6.3	Self-selected piece	Grand	EMG	Shoulder, upper limb (L)	Competition condition ↑ EMG activity and ↓ performance quality compared to no-competition condition	High (7)	3.7.3.
EMG	Kinematics	Kinetics	Multimodal						

A, amateurs; C, control; CP, concert pianists; E, experts; EMG, electromyography; LD, long duration group; L, left side only; LHS, large hand span; LL, loudness levels; MCP, metacarpophalangeal; MIDI, musical instrument digital interface; MP, mental practice; MSD, musculoskeletal disorders; N, novices; NP, normal practice; NR, not reported; PP, physical practice; R, right side only; RH, right hand only playing; ROM, range of motion; S/T, students/teachers; SD, short duration group; SHS, small hand span; TPT, “traditional playing” technique; VP, variable practice; WPT, “weight playing” technique.

### 2.4 Study risk of bias assessment

For each included study, two independent reviewers assessed the risk of bias following the Joanna Briggs Institute Critical Appraisal Checklist for analytical cross-sectional studies (Tufanaru et al., 2020). The possible answers were “yes,” “no,” “unclear,” or “not applicable.” A numeric score was assigned to each answer (i.e., “yes” = 1, else = 0). After the responses from both reviewers were compared and discussed, a summary score was calculated. Papers with four or fewer “yes” responses were classified as having a high risk of bias (i.e., poor quality), those with five or six “yes” responses as having a moderate risk of bias (i.e., average quality), and those with seven or eight “yes” responses as having a low risk of bias (i.e., high quality). The inter-rater reliability (Cohen’s Kappa) was computed after both reviewers (AJ, MP) had rated all the articles independently, and it indicated a strong degree of inter-rater agreement (*κ* = 0.89).

## 3 Results

**Figure 1:**
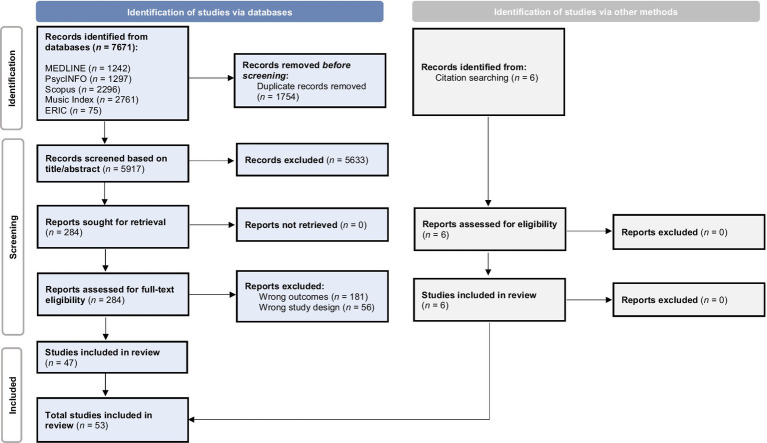
Selection of sources of evidence (PRISMA 2020 flow diagram).

**Figure 2:**
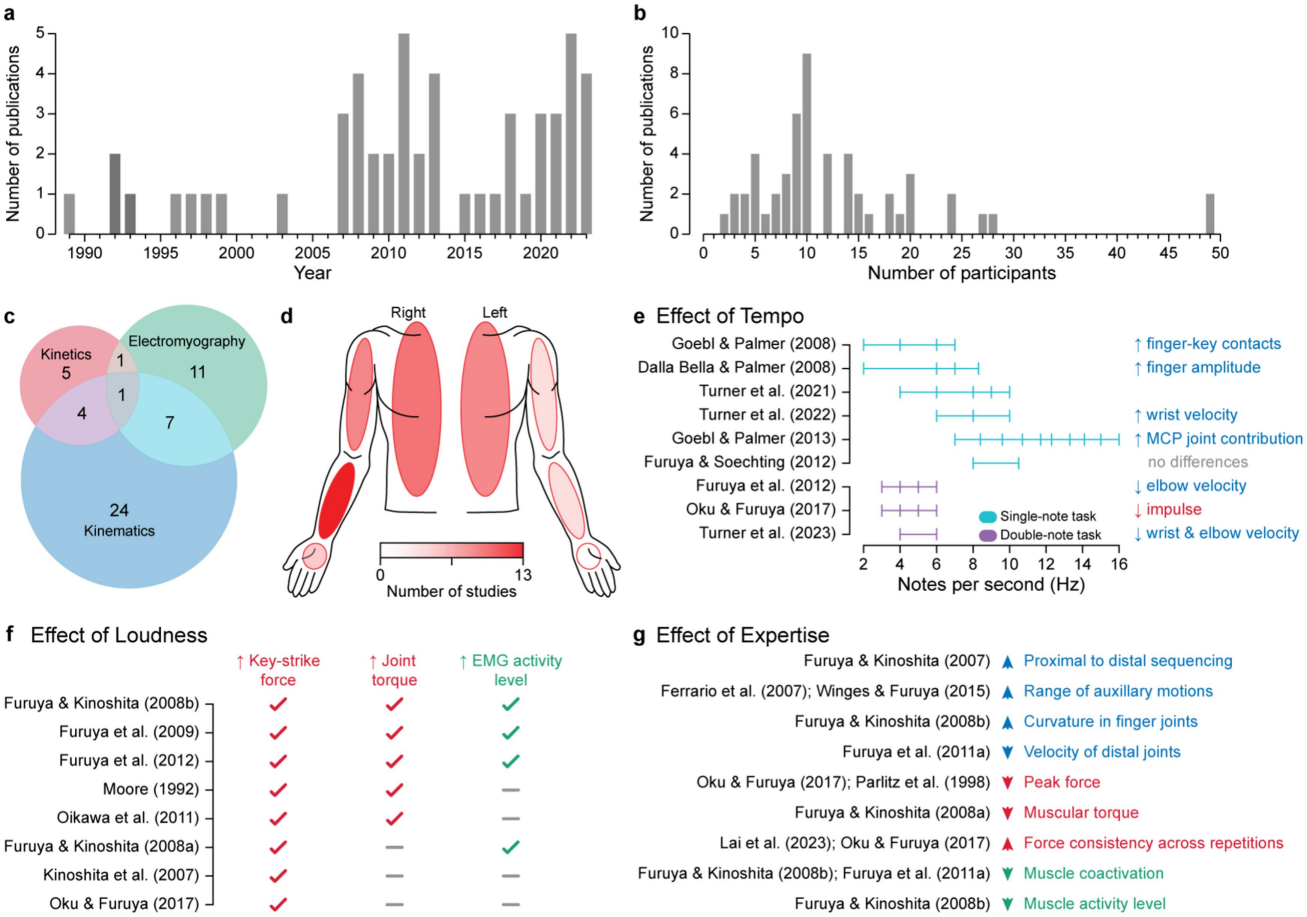
A synthesis of piano biomechanics literature. **(a)** Annual number of publications on piano biomechanics, showing a steady rise since 2007. **(b)** Distribution of study sample sizes in piano biomechanics. **(c)** Venn diagram showing an overview of the measurement types utilized across studies. Kinematics are most commonly measured, and interestingly, only one study out of the 53 used all three measurement modalities. **(d)** Body map showing the number of EMG investigations by body segment (hand, forearm, upper arm, and trunk). A clear right-side bias is evident when studying pianists. **(e)** A wide variety of tempi (keypresses / notes per second) have been investigated across studies. Primarily, two tasks—the faster single-note and the slower double-note task—were used when investigating biomechanical correlates of tempo changes in piano playing. Most of the reported effects of tempo are kinematic, with one exception. **(f)** The reported effect of loudness on kinetic parameters and EMG were consistent across all studies. **(g)** Summary of the effects of expertise.

A total of 7,671 studies were initially identified based on keyword searches of five electronic databases (Figure 1). Before screening, 1754 duplicates were removed, and 5,917 studies were screened based on the title and the abstract. In the following phase, 284 articles were assessed for full-text eligibility, of which 47 studies met the inclusion criteria. After identifying six additional studies through citation searching, the final number of studies included was 53. Regarding the risk of bias assessment, 62.3% of studies were classified as having a low risk of bias (i.e., high quality), 28.3% as having a moderate risk (i.e., average quality), and 9.4% as having a high risk of bias (i.e., low quality) (Supplementary Table S2). It is interesting to note that the first wave of publications on this topic occurred in the 1990s. After a brief gap in the early 2000s, research activity has steadily increased since 2007 (Figure 2a).

### 3.1 Sample characteristics

The number of participants ranged between two (Turner et al., 2021) and 49 (Goubault et al., 2021, 2023), with a median of 10 participants per study (Figure 2b). The included studies were conducted in eleven countries, including Japan (*n* = 14), USA (*n* = 11), Canada (*n* = 11), Italy (*n* = 6), Germany (*n* = 3), Belgium (*n* = 2), Taiwan (*n* = 2), and one each in Finland, Iran, Israel, and the United Kingdom. All of the studies involved advanced pianists, but the definition of skill varied since formal education is not a prerequisite for pianistic expertise. Therefore, participants in the highly trained group ranged from those with at least 10 years of piano lessons (e.g., Goebl and Palmer, 2013) to pianists holding or pursuing a doctoral degree in piano performance (e.g., Turner et al., 2023).

### 3.2 Task, tempo, and instrument characteristics

Given the inherent complexity of pianistic literature, obtaining uniform biomechanical measurements to answer specific research questions is challenging. Consequently, the included studies employed varied behavioral tasks, requiring participants to perform isolated keystrokes, octaves, or chords in a repetitive manner (*n* = 14), excerpts from the existing piano literature (*n* = 18), novel excerpts composed for the experiment (*n* = 8), self-selected excerpts from the participant’s repertoire (*n* = 5), or a combination of existing, novel, and self-selected excerpts (Table 2). In addition, one study included improvisation (Thio-Pera et al., 2022), one investigated isolated trills (Moore, 1992), two focused on scales (Sforza et al., 2003; Turner et al., 2021), and one study reported no specific details except for the duration of the task (Sugawara, 1999). Although playing with a metronome is uncommon in live performance contexts, many studies have used it to maintain control over the selected tempo. Specifically, 22 of the 53 studies included a metronome, five measured approximate trial-to-trial intervals for isolated keystrokes, four included the metronome or sound signals to cue the participant before the trial, 16 did not involve a metronome, and six provided no tempo instruction.

With regard to instruments used for the experiments, three options were utilized: a digital (*n* = 20), grand (*n* = 13), and upright (*n* = 13) piano, while seven studies did not disclose this information. In addition to biomechanical measurements, many studies employed MIDI-based data acquisition to capture keypress timing, velocity, and articulation parameters. MIDI data provide a fine-grained record of motor output by reflecting expressive and sequencing aspects of performance (van Vugt et al., 2014).

### 3.3 Measurements and anatomical regions

Out of the 53 papers reviewed, 24 studies utilized only kinematic measurements, five studies quantified the kinetic properties of movement, and 11 employed only EMG. Additionally, 13 studies involved combined measurements (EMG and kinematics, *n* = 7; EMG and kinetics, *n* = 1; kinematics and kinetics, *n* = 4), and one study employed all three methods (Furuya and Kinoshita, 2008b) (Figure 2c; Supplementary Table S3).

Regarding the anatomical regions, almost all articles focused on upper body movements (i.e., from the pelvis upwards), with the exception of Verdugo et al. (2020, 2022) who also examined the left lower limb. Furthermore, one study examined the trapezius muscles (Baeyens et al., 2022), two investigated lumbar erector spinae muscles (Honarmand et al., 2018; Aranceta-Garza et al., 2021), one focused solely on the left upper limb (Yoshie et al., 2009), and twelve investigated upper body bilaterally. The remaining 35 studies examined only right-sided upper limbs (in addition to occasional non-limb related parameters), as the right hand has the dominant role in piano repertoire in both melodic and virtuosic contexts (Figure 2d provides an overview of EMG measurement sites).

### 3.4 Influence of technique on kinematics, kinetics, and muscle activation

In a piano teaching context, “technique” has several meanings. It can refer to specific instructional methods like the Suzuki Method (Thibeault, 2018; Liu et al., 2024). It may also refer to postural guidelines for the wrist, fingers, shoulders, and spine during performance. Additionally, technique encompasses specialized exercises designed to develop finger independence, such as Hanon exercises (Hanon, 1873). For our purposes, we use technique to describe *pedagogical strategies* for executing musical elements like chords, scales, and adding nuance across different musical timeframes. These strategies include developing finger independence, refining touch quality, using selective relaxation, and employing the pedal. Since existing studies have extensively investigated types of touch and finger independence, we focused our review on how these two pedagogical strategies relate to biomechanical measurements.

#### 3.4.1 Type of touch

Pianistic technique involves different types of touch, either in the approach to the keyboard (struck versus pressed) or in the duration of the keypress (*staccato* versus *tenuto*), each influencing both the resulting sound and the underlying biomechanics. The importance of touch type is therefore multifaceted. Five studies investigated slow keystrokes produced with a single finger as a means to characterize the foundational properties of sound production on the piano. In these papers, the authors controlled for the type of touch by instructing participants to either initiate the descent toward the key-bottom from a self-selected height above the keys (struck touch) or with the fingertip already resting on the key surface (pressed touch). In these simple tasks, of particular interest was the investigation of intersegmental kinematic sequencing, which refers to the timing of peak angular velocities across joints involved in the movements that generated a key press (Putnam, 1993). When examining the intersegmental kinematics from the pelvis to the wrist, Verdugo et al. (2022) found proximal-to-distal sequencing during the pressed touch. In contrast, Furuya et al. (2010) identified the same temporal pattern in the struck touch only and observed distal-to-proximal sequencing for the pressed touch. This distal-to-proximal sequencing during the pressed touch was also characterized by lower elbow extension velocity compared to the struck touch, with compensation provided by increased shoulder and finger flexion velocities. An EMG study by Degrave et al. (2020) showed that the downward fingertip acceleration in the pressed touch was predominantly produced by the activity of the triceps brachii muscle. Additionally, this study found that *staccato* articulation (shorter keypresses) resulted in an increased burst of activity across almost all shoulder muscles compared to playing *tenuto* (longer keypresses). This was corroborated in a kinematic study by Verdugo et al. (2020) who found that *staccato* keystrokes produced 180 and 207% higher shoulder joint contribution to endpoint upward and forward velocities, respectively. Finally, Kinoshita et al. (2007) used kinetic measurements and found that the struck touch produced a higher initial force burst than the pressed touch, while also resulting in lower maximum force and impulse at the highest loudness levels, indicating that the struck touch is more biomechanically economical when playing loudly. However, they also found that the force developed more slowly during the pressed touch, thereby allowing pianists greater control of loudness modulation. Together, these findings demonstrate that the type of touch influences both biomechanical parameters and the resulting sound, highlighting the importance of choosing touch based on the demands of specific musical sections.

#### 3.4.2 Finger independence

While finger isolation is widely taught and practiced in pianistic pedagogy, there are inherent limits to how independently a finger can move due to finger coarticulation, a phenomenon wherein the movement of one finger is influenced by the movements of neighboring fingers due to shared muscles and tendons (Jerde et al., 2003). For instance, the flexor digitorum profundus muscle acts as a flexor of all four digits of the hand (Reilly and Schieber, 2003). In the context of piano playing, finger coarticulation primarily affects the execution of dexterous passages; ones that require rapid, interchangeable movements across fingers, unlike chords, which use more fixed hand positions. While coarticulation can facilitate smoother transitions between finger positions, it may also limit finger independence.

In the first of six studies that investigated finger independence, participants played excerpts that began with the same sequence and diverged after several keypresses (Engel et al., 1997). The results showed that pianists changed the movement patterns of non-striking fingers as much as 500 ms ahead of the last common note if a “thumb-under” maneuver was involved, indicating anticipatory adjustments. Furuya et al. (2011b) found that the overall movement coarticulation of the non-striking fingers was higher during thumb keystrokes compared to keystrokes made by other fingers. Winges et al. (2013) found that the distribution of EMG amplitudes across muscles varied depending on both the preceding and succeeding keypresses across a wide range of excerpts and Winges and Furuya (2015) showed that pianists employed a flexible strategy wherein the movement of non-striking fingers was sequence- and context-dependent. Additionally, Tominaga et al. (2016) found that the kinematic profile of the striking finger was primarily correlated with loudness consistency, while rhythmic consistency was impacted by both striking and non-striking fingers, indicating that finger coarticulation has a more pronounced effect on rhythmic precision.

Finger independence was also studied using kinetic measurements. In the seminal experiment by Parlitz et al. (1998), participants performed exercises where some fingers were constantly pressing a key while others were used alternately. The results showed that pianists maintained a low level of force for the key-holding fingers in two out of three tasks. However, in the most difficult of the three tasks, they demonstrated increased forces for the key-holding fingers, although there was no benefit in using more force than the minimum required to keep the keys depressed, indicating that the finger coarticulation impacts the force output even in fingers that are not actively moving. Together, these studies confirm that finger coarticulation persists even in pianists with sophisticated sensorimotor training. Nevertheless, pianists exhibit context-dependent adaptations to achieve greater flexibility in non-striking fingers and leverage finger coarticulation to their advantage.

### 3.5 Expertise

While experts unquestionably produce superior musical performances than novices, it remains unclear whether this stems from fundamentally different biomechanical strategies or from refined versions of the same basic approaches. In this review, we examine current literature on the biomechanical differences between experts and non-experts during comparable piano tasks that both groups can execute. A range of studies have compared the biomechanical strategies of expert pianists (typically developed through over 10,000 h of deliberate practice; Krampe and Ericsson, 1996) with those of less experienced players, either complete novices or amateur pianists. These comparisons span various tasks, including repeated octaves and sixths, broken intervals, and static key-hold exercises.

Furuya and Kinoshita (2007) found that experts employed proximal-to-distal intersegmental kinematics from the shoulder to the wrist when repeatedly playing an octave in a slow tempo, a temporal pattern that was not observed in novices. Subsequent studies by Furuya and Kinoshita (2008a, 2008b) further revealed that experts generated lower muscular torques at the elbow, wrist, and metacarpophalangeal joints and lower levels of coactivation of the finger muscles than novices. Additionally, experts utilized more curved finger positions, which resulted in smaller reaction forces at the metacarpophalangeal joint as the finger-joint centres were closer to the key-reaction-force vector. Furuya et al. (2009) found that experts also effectively utilized gravity during arm downswings by producing elbow extension and shoulder adduction through the deactivation of the anti-gravity activity of the biceps and deltoid muscles. Furthermore, Furuya et al. (2011a) showed that when participants played broken sixths in a tremolo pattern (rapid alternation between two notes), experts generated less flexion velocity at the thumb and little finger and more elbow pronation and supination velocity than novices. They also found that the fastest tempo was correlated with peak elbow velocity rather than finger velocity. Further studies explored differences in force control between experts and novices. Oku and Furuya (2017) found that expert pianists produced lower peak force and impulse across tempi and maintained more stable force output at slow tempi when playing repeated sixths. In a related study, Lai et al. (2023) showed that experts exhibited greater consistency in the fingertip force applied by the thumb and fifth finger, as well as smoother overall force profiles when playing repeated octaves.

Differences in the control of motion in non-striking fingers have also been documented. Ferrario et al. (2007) found that concert pianists exhibited greater displacement of non-striking fingers and more pronounced wrist excursions than piano teachers and students, indicating that advanced performers permit task-dependent residual motion. Similarly, when testing excerpts in which some fingers alternated between keys while others maintained a depressed key, Parlitz et al. (1998) reported that amateurs exerted greater forces in both striking and key-holding fingers. Finally, Winges and Furuya (2015) found that experts, unlike amateurs, selectively modulated non-striking finger movements based on musical context.

Across a range of pianistic tasks, expert performers demonstrate more consistent coordination, selective muscle activation, and flexible control of finger and joint motion compared to less-experienced players. A summary of these key biomechanical differences between expert and non-expert pianists is presented in Figure 2g.

### 3.6 Biomechanical adaptations to tempo, loudness, and expression

Due to the piano’s construction, pianists create musicality and expression by controlling the timing and loudness of hammer strikes, which are subtly modified by the pedals. Self-playing piano technologies demonstrate this principle by recreating performances through precisely replicating the timing and velocity of key presses and pedal movements (Suisman, 2010). Consequently, tempo (a timing element) and loudness represent the most thoroughly researched contextual parameters related to the musical score. In this section, we will review these parameters. We use the term “loudness” rather than “dynamics” (which musicians typically use) to avoid confusion with the biomechanical uses of the term dynamics (e.g., inverse dynamics, nonlinear dynamical systems).

#### 3.6.1 Effect of tempo

Nine studies examined the effect of tempo on movement patterns (Figure 2e). These studies used two tasks: a faster single-note task (6 studies) and a slower double-note task (3 studies). The examined tempi ranged from 2 to 16 notes per second. The number of tempi investigated in each study ranged from 2 to 10, with 4 being the most common. Most reported effects of tempo were kinematic.

Goebl and Palmer (2008) found that, as the tempo of simple melodies increased, participants produced more finger-key contacts, nearing 100% at the fastest tempo, thus increasing the proportion of struck touches. Since finger-key contacts can occur by initiating movement from various heights, Dalla Bella and Palmer (2011) tested vertical finger motion across different tempi and found that participants raised their fingers higher as the tempo increased. In a subsequent study, Goebl and Palmer (2013) instructed participants to play a five-tone melody at ten different tempi and showed that the metacarpophalangeal joint contributed most to the vertical fingertip motion, while individual joint contributions remained consistent across all tempi. In addition, across two tempi, Furuya and Soechting (2012) found no effect of tempo on kinematic consistency. However, it should be noted that the lowest rates in the latter two experiments were 7 and 8 notes per second, respectively, which corresponds to a moderately fast tempo rather than a slow one, as these rates align with moderate to fast ranges commonly found in classical repertoire. Broader tempo conditions were tested by Turner et al. (2021), who investigated motor strategies while participants played between 4 and 10 notes per second. They observed that changes in finger kinematics occurred at 8 notes per second; however, this study involved only two participants. In a subsequent study, Turner et al. (2022) examined three pianists with intentionally varied anthropometry and found that while variability in wrist kinematics increased with tempo, the observed differences were largely attributable to anatomical variation rather than tempo itself. Moreover, Turner et al. (2023) found that, during repetitive leap tasks at increasing tempi, greater trunk motion was associated with increased kinematic variability in the shoulder and elbow. Repetitive sixths were investigated by Furuya et al. (2012) across different tempi and loudness levels. As tempo increased, shoulder, wrist, and finger rotational velocities increased while elbow velocity decreased. Only the subgroup of international-competition winners increased both shoulder and wrist velocity while simultaneously reducing activity in the deltoid and extrinsic finger muscles. In a follow-up experiment, Oku and Furuya (2017) showed that differences in the maximum rate of repetitive keystrokes were explained by impulse rather than peak force, indicating that shorter durations of force per keypress enabled faster performance.

Together, these studies demonstrate that increasing tempo elicits systematic adjustments in joint coordination, finger trajectory, and segmental involvement, with additional strategies such as trunk motion and reduced impulse contributing to precision and efficiency at faster tempi (Figure 2e).

#### 3.6.2 Effect of loudness

Nine studies investigated loudness as a central determinant of biomechanical load in piano playing. Importantly, in scientific experiments direct control over loudness is more challenging than tempo: while tempo can be regulated precisely with the use of a metronome, loudness depends on subtle variations in force transfer through the key-hammer mechanism, making it a more challenging parameter to control. In these studies, loudness was typically cued in pianistic terms (e.g., *piano, forte*) while the resulting sound pressure level or key velocity was recorded to verify the outcome. Kinetic analyses showed that while participants maintained consistent key velocity across loudness levels, louder tones required disproportionately greater force and impulse, highlighting the energetic demands of loud playing (Kinoshita et al., 2007; Oku and Furuya, 2017). Moore’s EMG analysis of trills (Moore, 1992) similarly revealed that increases in loudness level raised the magnitude of forearm muscle activity, underscoring the added neuromuscular precision demanded at higher loudness. A study on octaves by Oikawa et al. (2011) showed that forearm muscle activity rose with loudness across all wrist positions, but the neutral wrist position minimized this increase. Further, Furuya and Kinoshita (2007) found that experts maintained the proximal-to-distal timing of peak angular velocities even as loudness increased, while Furuya and Kinoshita (2008b) demonstrated that muscle coactivation rose with loudness but less so in experts than novices. Furuya et al. (2009) likewise showed that experts increased biceps and triceps activity with greater loudness, *though to a smaller extent than novices*. Finally, Furuya and Kinoshita (2008a) showed that louder octaves amplify muscular and interaction torques at the elbow and wrist, while Furuya et al. (2012) found in repetitive sixths decreased elbow but increased shoulder, wrist, and finger velocities with greater muscular activity, together highlighting the compounding biomechanical demands of loudness control.

In sum, louder playing systematically heightens biomechanical load, expressed through increased force, torque, coactivation, and muscle activation, reflecting the compounded biomechanical demands of loudness control. Overall, the reported results on the effects of loudness were consistent across studies (Figure 2f).

#### 3.6.3 Effect of expression

Across four studies examining expressive and ancillary body movements, pianists consistently exhibited greater whole-body motion when performing expressively compared with non-expressive or deadpan conditions. Nakahara et al. (2010) showed that expressive playing elicited larger, rhythmically organized trunk and arm excursions that aligned with phrasing. Thompson and Luck (2012) and MacRitchie et al. (2013) further demonstrated that head and torso movements systematically mirrored musical structure, marking phrase boundaries and climaxes. Extending these findings, Massie-Laberge et al. (2019) quantified graded increases in head and torso motion across expressive conditions, identifying head movement as the most sensitive kinematic indicator of expressive intent. Collectively, these studies highlight the structural and communicative role of ancillary motion in pianistic expressivity. For a detailed analysis of sound-producing and sound-facilitating gestures, and a more focused review on musical expression, see Mailly et al. (2025).

### 3.7 Biomechanical measurements related to sustainable performance

Playing the piano professionally and sometimes even recreationally places substantial physical and mental demands on performers. Performing at a high level requires numerous daily hours of practice. A typical concert lasts 1–2 h, during which fatigue becomes a serious concern and ergonomic considerations grow increasingly important. The psychological stress of performing for extended periods before an audience adds another layer of challenge. Therefore, understanding both the helpful and harmful biomechanical adaptations to these demands can inform injury risk reduction strategies and help pianists sustain high performance levels throughout their careers. In this section, we review current literature on the biomechanical measurements related to sustainable performance.

#### 3.7.1 Muscular fatigue

Five studies assessed muscular fatigue using EMG median frequency, a commonly used indicator of fatigue (Marco et al., 2017). Goubault et al. (2021, 2023) used repetitive tasks sustained until participants reached a predefined level on the rate of perceived exertion scale and found a more pronounced decline in median frequency in wrist and finger extensors than in flexors. In addition, pianists who continued playing longer exhibited increased movement variability but less variability in EMG amplitude, demonstrating an adaptive redistribution of load. In contrast, two studies (Baeyens et al., 2020; McCrary et al., 2023) reported no clear evidence of muscular fatigue, whereas one study (Baeyens et al., 2022) observed a small decrease in EMG median frequency. The inconsistent findings regarding muscle fatigue during piano playing can be attributed to heterogeneity in research design. Baeyens et al. (2020, 2022) and McCrary et al. (2023) had participants perform various self-selected pieces without disclosing the musical details, except for total duration, which greatly limits the broader implications of their results. In contrast, Goubault et al. (2021, 2023) designed a fatiguing, repetitive piano task by asking participants to play short, intensive excerpts from works by Hanon and Liszt, while controlling for tempo and duration. Taken together, the conflicting results underscore how differences in task design and control conditions shape conclusions regarding fatigue in pianists. Relatedly, Furuya and Yokota (2018) demonstrated that rhythmic variation during practice reduced activity in intrinsic hand muscles, indicating that practice strategies can modulate muscular demand and potentially mitigate the development of fatigue.

#### 3.7.2 Ergonomics

Ergonomic factors such as bench design and hand span have also been studied. In an early study by Grieco et al. (1989), the lack of back support in standard piano benches was identified as a work-related risk. Honarmand et al. (2018) and Aranceta-Garza et al. (2021) confirmed that chairs with back support reduce erector spinae activation and improve comfort. However, both studies employed pianistically undemanding tasks, such as moderately fast scales and an intermediate-level Mozart sonata, which limits the generalizability of the findings. More demanding repertoire, such as Rachmaninov’s piano concertos, could benefit from greater effective mass generated by torso movements, which is not possible when resting against the backrest. In terms of anthropometrics, Lai et al. (2015) demonstrated that pianists with smaller hand spans used significantly more finger abduction and extension to reach larger intervals.

#### 3.7.3 Psychological stress

Finally, psychological stress has been studied in relation to its effects on muscle activation. Yoshie et al. (2008, 2009) found increased EMG amplitudes in performance-evaluation settings compared to non-evaluative practice conditions. Notably, only the pianist who performed best under pressure, as determined by a panel of judges in Yoshie et al. (2009), also exhibited reduced muscle activation compared to a non-evaluation condition, indicating individual variation in neuromuscular responses to stress.

Together, these studies on sustainable performance demonstrate that physical load in piano performance is shaped by the type of pianistic task, joint positioning, practice strategies, anatomical constraints, and psychological context. Finally, while fatigue-related changes were more pronounced in extensor muscles, clear evidence of muscular fatigue has only been observed in studies employing artificially fatiguing protocols and remains inconclusive under naturalistic performance and practice conditions.

## 4 Discussion

The purpose of this study was to examine existing research on the biomechanical basis of piano playing and synthesize the relationship between the pianistic technique and biomechanical measurements. Together, our findings indicate that touch type, finger independence, intersegmental kinematics, and muscle activation are modulated by pianists’ skill level, anthropometry, task demands, and muscular fatigue. In the following sections, we discuss and link these findings with practical implications for pedagogy, practice, and performance.

### 4.1 Technique-oriented practical implications of biomechanical research

Important implications can be drawn from existing studies on touch, which demonstrate that the struck touch is physiologically more efficient, particularly when playing loudly (see Section 3.4.1). Additionally, increased vertical finger motion associated with faster tempi correlates with higher temporal precision. Interestingly, this finding was also observed in clarinetists, where finger velocity does not affect loudness (Palmer et al., 2009), suggesting that increased vertical finger motion in pianists helps facilitate temporal precision despite the concurrent rise in loudness. However, this does not suggest that the struck touch is always superior. For example, the pressed touch is perceived as softer than the struck touch, largely due to the accompanying noise from the finger–key contact generated by the struck touch (Furuya et al., 2010). Furthermore, the pressed touch has a more gradual force profile, providing pianists with superior control of loudness levels. This is particularly important in softer sections, as lower sound levels demand greater force control (Kinoshita et al., 2007). In sum, while the struck touch is more efficient in generating force at the loudest dynamics in pieces that also require precise timing, the pressed touch is indispensable for delicately controlling the sound level in soft and slow lyrical passages. Overall, the contact between the finger and the piano key is a multimodal experience comprising auditory, visual, tactile, and proprioceptive components, all of which impact the decision on which type of touch to choose (Goebl, 2017). Understanding the physiological and artistic implications of different touch types can help pianists and pedagogues make informed decisions that enhance both performance quality and long-term physical health.

Existing studies related to finger independence demonstrate that pianists exhibit nuanced management of finger coarticulation, allowing for controlled movement of non-striking fingers rather than rigid isolation (see Section 3.4.2). Many established pedagogical traditions emphasize strict finger independence, exemplified by Hanon (1873), whose preface states: “If all five fingers of the hand were absolutely equally trained, they would be ready to execute anything written for the instrument, and the only question remaining would be that of fingering.” Although numerous pianistic schools have since emerged, the extreme finger independence and high-finger technique remain predominant in many regions, particularly in China (Xu, 2018). However, current biomechanical evidence highlights the benefits of leveraging natural muscular and neural coupling between fingers to achieve fluency and precision. Traditional teaching philosophies have also discouraged so-called “extraneous” movements, such as visible hand or finger motions unrelated to key depression, on the grounds that they waste energy or compromise control (e.g., Philipp, 1926). In contrast, recent biomechanical findings demonstrate that such anticipatory and non-striking movements can facilitate timing, coordination, and mechanical efficiency (Engel et al., 1997; Winges et al., 2013). Rather than discouraging all extraneous motion, educators should foster awareness of useful passive motion and emphasize smooth transitions across fingers. Cultivating this balance supports the development of finger independence without compromising biomechanical efficiency.

Finally, section 3.4.3 shows that expert pianists employ fundamentally different movement strategies than non-experts across a wide range of pianistic tasks. These include simple actions such as isolated octaves and sixths, movements that even novices can execute, as well as more complex sequences requiring fine motor control and coordination. Across these tasks, experts consistently exhibit optimized motor performance, including reduced muscle coactivation, more consistent force output, and coordinated proximal-to-distal sequencing of joint motion. There are two key takeaways from these findings. First, from a phenomenological standpoint, pianistic expertise emerges as a pervasive condition, evident even in tasks that do not require advanced musical training, highlighting the extraordinary complexity of piano playing as a skill. Second, the stark biomechanical differences between experts and non-experts underscore the importance of high-level, biomechanically-informed piano instruction from the earliest stages of training. Owing to the lack of institutional regulation, piano instruction is frequently delivered by underqualified teachers in many parts of the world, including the United States (Davidson and Jordan, 2007) and China (Zhu, 2018). The current evidence emphasizes that effective piano pedagogy must go beyond finger placement and note accuracy to include attention to the overall movement organization that facilitates long-term skill development.

### 4.2 Context-oriented practical implications of biomechanical research

Since the tempo and loudness of a piece are largely predetermined and only partly shaped by a pianist’s artistic intentions, their biomechanical effects are particularly relevant for practicing, which constitutes the vast majority of a pianist’s activities. Existing studies indicate that, at fast tempi, pianists do not simply perform the same motions faster. In dexterous passages, they utilize larger finger lifts, while in repetitive chordal sections, the entire organization of upper body movements changes. Similarly, studies on loudness show that higher loudness level is not achieved by a simple increase in force: Kinoshita et al. (2007) found disproportionately greater finger forces with limited acoustic gain, while Furuya and Kinoshita (2008b) highlighted the role of coordinated arm and wrist motions in producing efficient loud chords. These findings suggest that effective adjustments in both tempo and loudness require reorganizing movement patterns rather than merely scaling speed or force.

Although such results make it seem logical to practice at the final tempo, learning a new piece of music is a cognitively demanding process, and practicing immediately at fast tempi can lead to overlearned mistakes and maladaptive movement patterns. Indeed, Furuya et al. (2012) noted that as spatial accuracy demands increase with speed, skipping slower practice may impede the development of skillful multi-joint movement coordination. Practicing at a slower tempo naturally supports more accurate visual-motor integration in the early stages of learning. However, certain virtuosic passages should also be practiced at their final tempo from the outset, as this facilitates motor programming specific to the spatiotemporal characteristics of the pattern. In fact, Donald (1997) compared two different practice protocols with regard to tempo: (1) an incremental procedure, in which an excerpt was practiced at a slow, medium, and fast tempo, and (2) an alternate tempo protocol, with alternation between slow and fast tempi. The results showed that those following the alternate tempo protocol needed significantly fewer trials to achieve a specified speed and fluency, suggesting that an intermediate tempo does not enhance the rate of improvement. Thus, when the goal is to increase the speed while maintaining accuracy, practicing in very short segments with alternating slow and fast tempi might be more effective than practicing the entire section below the performance tempo, which remains a dominant strategy among musicians (Allingham and Wöllner, 2022). In addition, practicing with rhythmic variation, such as altering the relative timing of keypresses using dotted or swung rhythms, has been shown to be effective. Furuya and Yokota (2018) demonstrated that this approach led to equivalent performance improvements as practicing without rhythms while reducing muscle activity, supporting its use as an efficient practice strategy to bridge the gap between slow and fast playing.

Included studies also demonstrate that both physiological and psychological stress are accompanied by increased muscle activity, which can negatively affect the control of loudness and timing (e.g., Goubault et al., 2021; Yoshie et al., 2009). To limit physical stress and prevent the overload of specific muscles and tendons, pianists can alternate between different movement configurations (e.g., chords and dexterous passages), rather than repeating a single pattern for extended periods. Additionally, caution is advised with excessive *staccato* practice (Degrave et al., 2020) and with passages that are both loud and fast (Furuya et al., 2012), as these have been linked to increased muscular demand. One potential mitigation strategy is using curved finger positions to distribute the reaction forces from the keys more efficiently (Furuya and Kinoshita, 2008b).

Despite the significant variability in hand span among pianists (Wagner, 1988), the size of the piano keyboard has remained largely uniform since the second half of the 19th century (Sakai, 2008). Pianists with smaller hand spans may encounter repertoire limitations (Deahl and Wristen, 2017), experience increased muscle activity (Chi et al., 2021), and endure greater stress due to having smaller joints and tendons, as the same force is distributed over a smaller area (Wolf et al., 1993). In such cases, the authors recommend freely arpeggiating wide chords, as the resulting musical compromise is minor compared to the physical risks posed by forced extensions and abductions. Fatigue-related adaptations should also be considered: since some pianists adjust their movement strategies as fatigue accumulates, increasing variability to reduce localized load can help sustain performance.

One of the most psychologically demanding aspects of piano playing is delivering performances on stage (Kirchner, 2003). Unlike many instrumentalists, pianists frequently perform solo and are expected to memorize structurally complex music (Chaffin and Imreh, 2002). Desensitization through repeated exposure may be beneficial; Steptoe (1989) found that more experienced musicians showed lower levels of anxiety than students, both cognitively and physiologically. When formal performing opportunities are limited, teachers can simulate performance conditions through peer run-throughs or studio classes. Although performance anxiety and injury risk were not the primary focus of this review (see Baadjou et al., 2016; Stanhope et al., 2022), understanding the musculoskeletal responses to stress can help inform more sustainable and effective practice routines. Ultimately, developing refined control over muscle activation by minimizing unnecessary load and distributing effort efficiently is essential both for pianistic virtuosity and for reducing the risk of injury.

While our review focused on expert pianists, recent work has used piano performance as a model for motor learning in naïve participants (Paolini et al., 2024), suggesting that expert pianist biomechanics may represent an asymptotic target of skill acquisition. This perspective links performance science with broader questions of motor learning, neuroplasticity, and rehabilitation, emphasizing how piano training paradigms can serve as controlled yet ecologically valid frameworks for studying skill acquisition and sensorimotor adaptation.

### 4.3 Limitations

When interpreting the findings from this review, several limitations should be noted. The selected themes reflect a pianistically informed grouping of the existing biomechanical literature. While they are unquestionably valuable to pianists (some of the authors are active concert pianists), they do not represent a comprehensive map of all relevant topics. Furthermore, ten studies could not be assigned to a thematic group due to either unique focus or insufficient methodological detail. Consistent with PRISMA guidelines, they are presented in the summary table but excluded from the thematic synthesis. Important areas remain underexplored, such as pedalling coordination, fatigue over long performances, hand-span adaptations, and more nuanced biomechanics of octaves and chords. More broadly, current research often isolates specific components of technique, whereas pianists engage with the instrument in a highly integrated and expressive manner. Future work would benefit from addressing the holistic nature of piano playing and how technical elements interact in real performance contexts.

The majority of the studies recruited only right-handed participants and measured only the right-sided upper limbs. While this approach results in a more scientifically controlled environment, limited conclusions can be drawn regarding the impact of lateralization and the extent to which these findings apply to more complex tasks commonly encountered in piano repertoire. Furthermore, considering that the piano repertoire includes far more virtuosity for the right hand and the general predominance of right-handers, this results in different proficiency levels between the hands, which has only been quantified in terms of MIDI data so far (Kim et al., 2021; Van Vugt and Altenmüller, 2019). Moreover, making pedal adjustments is an essential component of almost all piano pieces, yet the pedaling technique remains biomechanically unexplored. Concerning the choice of the instrument, digital pianos were the most frequently utilized across studies despite their reduced touch and sound quality compared to acoustic pianos. However, the recent development of hybrid pianos, which utilize the mechanism of an acoustic piano in a smaller frame, might help balance the trade-off between the size and quality of the instruments used in research.

The included papers focused on muscular load and fatigue, therefore the microstructural damage that occurs in tendons, such as in lateral epicondylitis, is not well understood despite being one of the most frequent injuries among pianists (Goubault et al., 2021). In general, there have been a significant number of publications investigating focal dystonia, a rare neurological disorder with a prevalence rate of 1–2% among musicians (e.g., Furuya and Oku, 2023); still, direct research on the etiology of the more common tendon-related injuries is lacking. Furthermore, the studies on injuries lack biomechanical measurements of the injuries themselves, instead relying on questionnaires (e.g., Bruno et al., 2008).

Some limitations can be attributed to the nature of existing biomechanical measurements. For example, while optical or camera-based motion capture systems are considered the gold standard for precision, they involve marker placements, which can be burdensome when investigating fine motor control, such as in piano playing. Recent studies have introduced markerless motion capture systems employing machine learning algorithms, which are in some cases as accurate and reliable as marker-based systems (e.g., Kanko et al., 2021) and can minimize the risk of measurement-induced artifacts (Armitano-Lago et al., 2022). While these high-precision markerless systems have not yet been optimized for smaller joints, such as fingers, they present exciting future opportunities in performance science.

## 5 Conclusion and future directions

Piano playing involves an intricate interplay of the cognitive, neuromuscular, and musculoskeletal systems, wherein the performer’s body is a necessary medium between the musical idea and the musical instrument. Current research has shed light on aspects of piano playing that cannot be explained by tradition and intuition alone. For example, Furuya et al. (2012) found that increasing tempo led to a decrease in elbow flexion velocity even as other joint velocities increased, which contradicts intuitive expectations of uniformly faster movement and would be difficult to detect without motion capture. Similarly, as sound intensity (dB) increases approximately linearly, the keystroke force required to produce those tones rises exponentially, indicating an inefficiency in force production not apparent from auditory feedback alone. By synthesizing findings from 53 studies across more than three decades, this review provides a unified resource for both researchers and practitioners, bridging gaps between scientific insights and pedagogical practice. Beyond pedagogy and physiology, piano biomechanics also offers insight into how bodily coordination shapes expression and communication in performance. For example, differences in joint sequencing and load distribution can modify tone quality and nuances in loudness, revealing how mechanical adjustments translate into expressive outcomes. However, the majority of investigations into the biomechanical basis of piano playing have focused on simple, repetitive motions. Instead, examining musically meaningful sections and movement patterns would more effectively translate scientific insights into teaching methods. While there is an accumulating wealth of biomechanical data involving piano playing, we are yet to come up with a unifying theory of piano playing that could inform practice. For instance, teaching instructions typically favor specific anthropometric features, and we envision that a mechanistic theory of piano playing would provide a systematic framework for adapting existing pedagogy to individualized instruction. In addition, longitudinal studies would play a complementary role in specifying maladaptive processes leading to performance-related injuries. Therefore, deeper interdisciplinary collaborations between piano practitioners and scientists are needed to develop a mechanistic understanding of piano playing, translate these insights into teaching methods, and promote wellbeing by minimizing the risk of performance-related injuries.

## Data Availability

The original contributions presented in the study are included in the article/Supplementary material, further inquiries can be directed to the corresponding authors.
