# Supplementary material for: The biomechanics of piano playing: a systematic review of kinematic, kinetic, and electromyographic literature
Source: Front Psychol. 2026 Jan 5;16:1690422. doi: 10.3389/fpsyg.2025.1690422 (PMC12812707; doi:10.3389/fpsyg.2025.1690422)
Supplement: Supplementary file 1 [file Table_1.DOCX]

**Table S1. Full electronic search strategy.**

| **Database** | **MEDLINE** (Ovid) | | |
| --- | --- | --- | --- |
| **Date** | January, 2024 | | |
| **Limits** | Language: “English”, Publication Type: “Article” | | |
| **Search Query** | **Population, Exposure** | **AND** | **Outcomes** |
|  | ***Pianists / Piano Playing*** |  | ***Biomechanics*** |
|  | ***Keywords***  piano.mp. OR  (music* adj3 perform*).mp. OR pianist*.mp. |  | ***Keywords***  (biomechanic* or movement* or muscle* or electromyograph* or motor* or limb* or motion* or mechanic* or kinematic* or kinetic*).mp |
| **Results** | 1242 | | |

| **Database** | **PsycInfo** | | |
| --- | --- | --- | --- |
| **Date** | January, 2024 | | |
| **Limits** | Language: “English”, Publication Type: “Article” | | |
| **Search Query** | **Population, Exposure** | **AND** | **Outcomes** |
|  | ***Pianists / Piano Playing*** |  | ***Biomechanics*** |
|  | ***Keywords***  (piano or (music* adj3 perform*) or pianist*).mp. |  | ***Keywords***  (biomechanic* or movement* or muscle* or electromyograph* or motor* or limb* or motion* or mechanic* or kinematic* or kinetic*).mp. |
| **Results** | 1297 | | |

| **Database** | **SCOPUS** | | |
| --- | --- | --- | --- |
| **Date** | January, 2024 | | |
| **Limits** | Language: “English”, Publication Type: “Article” | | |
| **Search Query** | **Population, Exposure** | **AND** | **Outcomes** |
|  | ***Pianists / Piano Playing*** |  | ***Biomechanics*** |
|  | ***Keywords***  (piano OR pianist) |  | ***Keywords***  (biomechanic* OR movement* OR muscle* OR electromyograph* OR motor* OR limb* OR motion* OR mechanic* OR kinematic* OR kinetic*) |
| **Results** | 2296 | | |

| **Database** | **Music Index** | | |
| --- | --- | --- | --- |
| **Date** | January, 2024 | | |
| **Limits** | Language: “English”, Publication Type: “Article” | | |
| **Search Query** | **Population, Exposure** | **AND** | **Outcomes** |
|  | ***Pianists / Piano Playing*** |  | ***Biomechanics*** |
|  | ***Keywords***  (piano or (music* adj3 perform*) or pianist*) |  | ***Keywords***  (biomechanic* or movement* or muscle* or electromyograph* or motor* or limb* or motion* or mechanic* or kinematic* or kinetic*) |
| **Results** | 2761 | | |

| **Database** | **ERIC** | | |
| --- | --- | --- | --- |
| **Date** | January, 2024 | | |
| **Limits** | Language: “English”, Publication Type: “Article” | | |
| **Search Query** | **Population, Exposure** | **AND** | **Outcomes** |
|  | ***Pianists / Piano Playing*** |  | ***Biomechanics*** |
|  | ***Keywords***  (piano or pianist*) |  | ***Keywords***  (biomechanic* or movement* or muscle* or electromyograph* or motor* or limb* or motion* or mechanic* or kinematic* or kinetic*) |
| **Results** | 75 | | |

**Table S2. Risk of bias assessment of all studies included in the review.**

Q1: Were the criteria for inclusion in the sample clearly defined?

Q2: Were the study subjects described in detail?

Q3: Was the setting/instrument described in detail?

Q4: Were controls involved in the study?

Q5: Was the task/excerpt suitably aligned with the study's objectives?

Q6: Were the outcomes measured in a valid and reliable way?

Q7: Was appropriate statistical analysis used?

Q8: Was a clear discussion considering implications and limitations presented in the article?

High risk of bias (i.e., poor quality) = 4 or fewer "yes" responses

Moderate risk of bias (i.e., average quality) = 5 or 6 "yes" responses

Low risk of bias (i.e., high quality) = 7 or more “yes" responses

| **Author (Year)** | **Q1** | **Q2** | **Q3** | **Q4** | **Q5** | **Q6** | **Q7** | **Q8** | **Risk of Bias**  (# of "yes" responses) |
| --- | --- | --- | --- | --- | --- | --- | --- | --- | --- |
| Aranceta-Garza et al. (2021) | Y | Y | Y | N | Y | Y | Y | Y | Low (7) |
| Baeyens et al. (2020) | Y | Y | N | N | Unclear | Y | Y | Y | Moderate (5) |
| Baeyens et al. (2022) | Y | Y | N | N | Unclear | Y | Y | Y | Moderate (5) |
| Bernardi et al. (2013) | Y | Y | Y | Y | Y | Y | Y | Y | Low (8) |
| Chung et al. (1992) | Unclear | Y | Y | N | Y | Y | Unclear | N | High (4) |
| Dalla Bella & Palmer (2011) | Y | Y | Y | N | Y | Y | Y | Y | Low (7) |
| Degrave et al. (2020) | Y | Y | Y | N | Y | Y | Y | Y | Low (7) |
| Engel et al. (1997) | Y | N | Y | N | Y | Y | Y | Unclear | Moderate (5) |
| Ferrario et al. (2007) | Y | Y | Y | Y | Unclear | Unclear | Y | Y | Moderate (6) |
| Furuya & Kinoshita (2007) | Y | Y | Y | Y | Y | Y | Y | N | Low (7) |
| Furuya & Kinoshita (2008a) | Y | Y | Y | Y | Y | Y | Y | Unclear | Low (7) |
| Furuya & Kinoshita (2008b) | Y | Y | Y | Y | Y | Y | Y | Unclear | Low (7) |
| Furuya et al. (2009) | Y | Y | Y | Y | Y | Y | Y | Unclear | Low (7) |
| Furuya et al. (2010) | Y | Y | Y | N | Y | Y | Y | Unclear | Moderate (6) |
| Furuya et al. (2011a) | Y | Y | Y | Y | Y | Y | Y | Y | Low (8) |
| Furuya et al. (2011b) | Y | Y | Y | N | Y | Y | Y | Unclear | Moderate (6) |
| Furuya et al. (2012) | Y | Y | Y | N | Y | Y | Y | Unclear | Moderate (6) |
| Furuya & Soechting (2012) | Y | Y | Y | N | Y | Y | Y | Y | Low (7) |
| Furuya & Yokota (2018) | Y | Y | Y | Y | Y | Y | Y | Unclear | Low (7) |
| Goebl & Palmer (2008) | Y | Y | Y | N | Y | Y | Y | Y | Low (7) |
| Goebl & Palmer (2013) | Y | Y | Y | N | Y | Y | Y | Y | Low (7) |
| Goubault et al. (2021) | Y | Y | Y | N | Y | Y | Y | Y | Low (7) |
| Goubault et al. (2023) | Y | Y | Y | N | Y | Y | Y | Y | Low (7) |
| Grieco et al. (1989) | Y | N | Y | N | Y | Y | N | Y | Moderate (5) |
| Honarmand et al. (2018) | Y | Y | N | N | Unclear | Y | Y | Unclear | High (4) |
| Kaufman-Cohen et al. (2018) | Y | Y | N | N | Y | Y | Y | Y | Moderate (6) |
| Kinoshita et al. (2007) | Y | Y | Y | N | Y | Y | Y | Y | Low (7) |
| Lai et al. (2015) | Y | Y | Y | Y | Y | Y | Y | Y | Low (8) |
| Lai et al. (2023) | Y | Y | Y | Y | Y | Y | Y | Y | Low (8) |
| MacRitchie et al. (2013) | Y | Y | Y | N | Y | Y | Y | Y | Low (7) |
| Massie-Laberge et al. (2019) | Y | Y | Y | N | Y | Y | Y | Y | Low (7) |
| McCrary et al. (2023) | Y | Y | N | N | Unclear | Y | Y | Y | Moderate (5) |
| Moore (1992) | Y | N | Y | N | Y | Y | Unclear | Unclear | High (4) |
| Nakahara et al. (2011) | Y | Y | Y | N | Y | Y | Y | Y | Low (7) |
| Oikawa et al. (2011) | Y | Y | Y | Y | Y | Y | Y | Unclear | Low (7) |
| Oku & Furuya (2017) | Y | Y | Y | Y | Y | Y | Y | Y | Low (8) |
| Parlitz et al. (1998) | Y | Y | Y | Y | Y | Y | Y | Unclear | Low (7) |
| Sakai et al. (1996) | Y | Y | Y | Y | Y | Y | Unclear | Unclear | Moderate (6) |
| Sforza et al. (2003) | Y | Y | Y | N | Y | Y | Y | Y | Low (7) |
| Sugawara (1999) | Y | N | N | N | N | Y | Unclear | Y | High (3) |
| Thio-Pera et al. (2022) | Y | Y | Y | N | Y | Y | Y | Y | Low (7) |
| Thompson & Luck (2011) | Y | Y | Y | N | Y | Y | Y | Y | Low (7) |
| Tominaga et al. (2016) | Y | Y | Y | N | Y | Y | Y | Y | Low (7) |
| Turner et al. (2021) | Y | N | Y | N | Y | Y | Y | Y | Moderate (6) |
| Turner et al. (2022) | Y | N | Y | N | Y | Y | Y | Y | Moderate (6) |
| Turner et al. (2023) | Y | Y | Y | N | Y | Y | Y | Y | Low (7) |
| Verdugo et al. (2020) | Y | Y | Y | N | Y | Y | Y | Y | Low (7) |
| Verdugo et al. (2022) | Y | Y | Y | N | Y | Y | Y | Y | Low (7) |
| Winges et al. (2013) | Y | Y | Y | Y | Y | Y | Y | Unclear | Low (7) |
| Winges & Furuya (2015) | Y | Y | Y | Y | Y | Y | Y | Unclear | Low (7) |
| Wolf et al. (1993) | Y | N | Y | N | Y | N | Y | Y | Moderate (5) |
| Yoshie et al. (2008) | N | Y | Y | N | Y | Y | Y | Unclear | Moderate (5) |
| Yoshie et al. (2009) | Y | Y | Y | N | Y | Y | Y | Y | High (7) |

**Table S3. Data collection tools.**

| **AUTHOR(S)** | **KINEMATICS** | **EMG** | **KINETICS** |
| --- | --- | --- | --- |
| Aranceta-Garza et al. (2021) | - | High-density | - |
| Baeyens et al. (2020) | - | Bipolar | - |
| Baeyens et al. (2022) |  | Bipolar |  |
| Bernardi et al. (2013) | 3D motion capture | - | - |
| Chung et al. (1992) | Biaxial electrogoniometer | - | - |
| Dalla Bella & Palmer (2011) | 3D motion capture | - | - |
| Degrave et al. (2020) | 3D motion capture | Bipolar | - |
| Engel et al. (1997) | 2D motion capture | - | - |
| Ferrario et al. (2007) | 3D motion capture | - | - |
| Furuya & Kinoshita (2007) | 2D motion capture | - | - |
| Furuya & Kinoshita (2008a) | 2D motion capture | - | Force transducer |
| Furuya & Kinoshita (2008b) | 2D motion capture | Bipolar | - |
| Furuya et al. (2009) | - | Bipolar | Force transducer |
| Furuya et al. (2010) | 2D motion capture | - | Force transducer |
| Furuya et al. (2011a) | 3D motion capture | Bipolar | - |
| Furuya et al. (2011b) | 3D motion capture | - | - |
| Furuya et al. (2012) | 2D motion capture | Bipolar | - |
| Furuya & Soechting (2012) | Data glove | - | - |
| Furuya & Yokota (2018) | - | Bipolar | - |
| Goebl & Palmer (2008) | 3D motion capture | - | - |
| Goebl & Palmer (2013) | 3D motion capture | - | - |
| Goubault et al. (2021) | - | High-density | - |
| Goubault et al. (2023) | Inertial measurement units | High-density |  |
| Grieco et al. (1989) | - | Bipolar | - |
| Honarmand et al. (2018) | - | Bipolar | - |
| Kaufman-Cohen et al. (2018) | 3D motion capture | - | - |
| Kinoshita et al. (2007) | - | - | Force transducer |
| Lai et al. (2015) | 3D motion capture | - | Force transducer |
| Lai et al. (2023) | - | - | Force transducer |
| MacRitchie et al. (2013) | 3D motion capture | - | - |
| Massie-Laberge et al. (2019) | 3D motion capture | - | - |
| McCrary et al. (2023) | - | Bipolar | - |
| Moore (1992) | Uniaxial accelerometer | Bipolar | - |
| Nakahara et al. (2010) | 3D motion capture | - | - |
| Oikawa et al. (2011) | Uniaxial electrogoniometer | Bipolar | - |
| Oku & Furuya (2017) | - | - | Force transducer |
| Parlitz et al. (1998) |  |  | Pressure sensor matrix |
| Sakai et al. (1996) | 3D motion capture | - | - |
| Sforza et al. (2003) | 3D motion capture | - | - |
| Sugawara (1999) | Biaxial electrogoniometer | - | - |
| Thio-Pera et al. (2022) | 3D motion capture | High-density | - |
| Thompson & Luck (2011) | 3D motion capture | - | - |
| Tominaga et al. (2016) | 3D motion capture | - | - |
| Turner et al. (2021) | 3D motion capture | - | - |
| Turner et al. (2022) | 3D motion capture | - | - |
| Turner et al. (2023) | 3D motion capture | - | - |
| Verdugo et al. (2020) | 3D motion capture | - | - |
| Verdugo et al. (2022) | 3D motion capture | - | - |
| Winges et al. (2013) | - | Bipolar | - |
| Winges & Furuya (2015) | Data glove | - | - |
| Wolf et al. (1993) | - | - | Inverse dynamics |
| Yoshie et al. (2008) | - | Bipolar | - |
| Yoshie et al. (2009) | - | Bipolar | - |
